# Consideration of Sex as a Biological Variable in the Development of Doxorubicin Myotoxicity and the Efficacy of Exercise as a Therapeutic Intervention

**DOI:** 10.3390/antiox10030343

**Published:** 2021-02-25

**Authors:** Ryan N. Montalvo, Vivian Doerr, Branden L. Nguyen, Rachel C. Kelley, Ashley J. Smuder

**Affiliations:** Department of Applied Physiology and Kinesiology, University of Florida, Gainesville, FL 32611, USA; ryan.montalvo@ufl.edu (R.N.M.); vdoerr@ufl.edu (V.D.); branden.nguyen@ufl.edu (B.L.N.); kelleyrc@ufl.edu (R.C.K.)

**Keywords:** skeletal muscle, cardiac muscle, chemotherapy, cancer, sexual dimorphism

## Abstract

Doxorubicin (DOX) is an anthracycline antibiotic used to treat a wide variety of hematological and solid tumor cancers. While DOX is highly effective at reducing tumor burden, its clinical use is limited by the development of adverse effects to both cardiac and skeletal muscle. The detrimental effects of DOX to muscle tissue are associated with the increased incidence of heart failure, dyspnea, exercise intolerance, and reduced quality of life, which have been reported in both patients actively receiving chemotherapy and cancer survivors. A variety of factors elevate the probability of DOX-related morbidity in patients; however, the role of sex as a biological variable to calculate patient risk remains unclear. Uncertainty regarding sexual dimorphism in the presentation of DOX myotoxicity stems from inadequate study design to address this issue. Currently, the majority of clinical data on DOX myotoxicity come from studies where the ratio of males to females is unbalanced, one sex is omitted, and/or the patient cohort include a broad age range. Furthermore, lack of consensus on standard outcome measures, difficulties in long-term evaluation of patient outcomes, and other confounding factors (i.e., cancer type, drug combinations, adjuvant therapies, etc.) preclude a definitive answer as to whether differences exist in the incidence of DOX myotoxicity between sexes. This review summarizes the current clinical and preclinical literature relevant to sex differences in the incidence and severity of DOX myotoxicity, the proposed mechanisms for DOX sexual dimorphism, and the potential for exercise training to serve as an effective therapeutic countermeasure to preserve muscle strength and function in males and females.

## 1. Introduction

Doxorubicin (i.e., Adriamycin) is a highly effective anti-tumor agent used primarily in the treatment of hematological malignancies and solid tumor cancers, including but not limited to leukemia, lymphoma, and cancer of the breast, stomach, lung, ovaries, and thyroid. Unfortunately, the use of doxorubicin (DOX) is limited due to the development of toxic side effects within both cardiac and skeletal muscle [1]. Analysis of DOX distribution within patients and animals has identified greater accumulation within highly oxidative tissues, including the heart and skeletal muscle, potentially due to their high vascularization and cardiolipin content [2,3,4]. Localization of DOX to cardiolipin within the inner mitochondrial membrane disrupts redox balance, resulting in myofibrillar protein oxidation and breakdown of the contractile apparatus [5,6,7,8].

While there are several established risk factors for DOX-induced myopathy, including dose, age, and comorbidity, there are currently limited data comparing off-target toxic outcomes between the sexes [9,10]. In this regard, determination of the appropriate balance between antineoplastic efficacy and cytotoxicity, and establishment of the maximum tolerated dose of chemotherapy drugs, historically does not consider the potential impact of sex [11]. A major factor contributing to this omission was that prior to 1993, the United States Food and Drug Administration (FDA) restricted the participation of women with childbearing potential in Phase 1 and early Phase 2 trials. Published FDA Guidelines for the Study and Evaluation of Gender Differences in the Clinical Evaluation of Drugs (58 FR 39406) along with the National Institutes of Health 2015 Policy on Sex as a Biological Variable (NOT-15-102) have attempted to rectify the earlier exclusion. Accordingly, progress needs to be made in addressing sexual dimorphic responses to DOX treatment, with a primary outcome the development of effective therapeutic countermeasures to combat toxicity.

This report will provide systematic characterization of sex-based differences related to DOX cytotoxicity, with specific emphasis on cardiac and skeletal muscle. We begin with an overview of evidence of sex disparities in the presentation of DOX-induced muscle toxicity in both clinical and preclinical reports, followed by discussion of variability in the mechanisms reported to promote DOX myotoxicity. Finally, based on the perceived efficacy of exercise training to mitigate cardiorespiratory dysfunction and fatigue following DOX treatment, we will provide a critical analysis of the effectiveness of exercise to offer protection between the sexes.

## 2. Doxorubicin-Induced Myotoxicity

**Figure 1:**
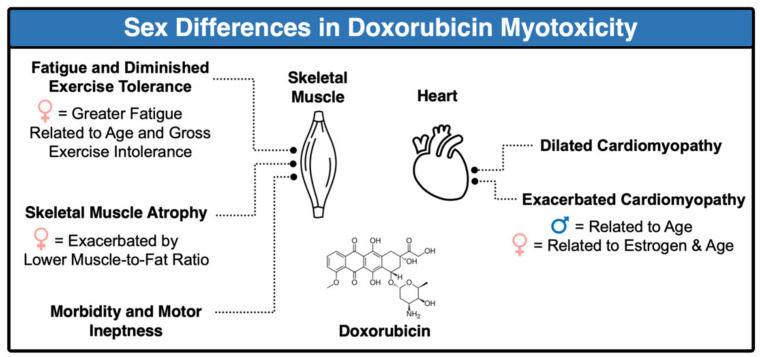
Sex differences in doxorubicin-induced cardiac and skeletal muscle toxicity.

Anthracyclines are a class of chemotherapy agent that are widely utilized and highly effective at reducing cancer tumor burden, with DOX among the most commonly used in clinical practice [12]. The mode of action for DOX’s antineoplastic activity includes (1) DNA intercalation, (2) topoisomerase II inhibition, and (3) the generation of free radicals [13]. These effects promote tumor cell death by enhancing DNA damage, disrupting DNA synthesis, and preventing DNA double strand recombination. However, repeated exposure to DOX can affect cellular apoptosis, leading to DOX resistant tumor cells and increased myotoxicity [13]. In this section, we will review clinical and preclinical evaluations of sex differences that exist in the incidence of DOX-induced cardiac and skeletal muscle pathology (Figure 1).

### 2.1. Clinical Manifestation of Doxorubicin Cardiotoxicity

The cardiotoxic effects of DOX are traditionally categorized by the time of onset following DOX exposure [14]. Acute cardiotoxicity is defined as transient cardiac events that can occur immediately after treatment or within the first days to weeks [15]. Symptoms develop as a result of cardiac edema and inflammation, and include cardiac arrhythmia, decreased contractility, hypotension, pericarditis, and myocarditis [16,17,18]. Early-onset chronic cardiotoxicity appears within one year after the completion of DOX treatment, the clinical presentation of which varies based on patient age [15]. Adult patients most commonly develop dilated cardiomyopathy, characterized by thinning of the ventricle wall and increased ventricle size. In contrast, pediatric patients present with dilated cardiomyopathy that progressively transitions to restrictive cardiomyopathy [14,19]. Finally, late-onset chronic cardiotoxicity can develop years or even decades after the end of chemotherapy [20], with patients that become symptomatic displaying dilated or restrictive cardiomyopathy [16,21].

#### 2.1.1. Sex-Related Differences in the Development of Cardiotoxicity: Clinical Perspective

DOX-induced cardiac dysfunction was established during early clinical trials, in which a dose-dependent development of electrophysiological abnormalities and congestive heart failure (CHF) was observed in cancer patients [22]. Investigation into the etiology of DOX cardiotoxicity has identified risk factors associated with increased incidence of adverse cardiac events (i.e., advancing age, comorbidities, dose, etc.) [15]; however, the role of sex as a risk factor remains unclear. In general, defining sex differences in the prevalence of heart failure has been limited due to many studies’ failure to perform statistical comparisons between males and females, underrepresentation of one sex, and/or inability to make accurate comparisons between patients with different types of cancer or treatment history. One of the first studies to consider sex in the development of cardiomyopathy in patients receiving DOX evaluated 1273 patients to determine factors other than dose that predicted the incidence of cardiomyopathy [23]. In this diverse cohort of patients, the occurrence of cardiomyopathy was higher in female subjects compared to males, but no statistical significance existed [23]. Later, Hrushesky and colleagues reported differences between males and females in the development of DOX-induced CHF [24]. This study revealed that females developed CHF at a greater rate when receiving an equivalent average dose of DOX to males. While this finding was novel, the interpretation was confounded by the imbalance between males and females, the wide age range, and disparity in cancer type [24].

Subsequent studies have attempted to limit patient variability by evaluating sex differences by age at diagnosis. In pediatric cancer survivors, significant trends toward higher female risk were first established [25,26]. However, as more studies emerge highlighting relative risk between sexes in the pediatric cancer patient population, there is no clear consensus, since greater risk for females [27,28], greater risk for males [29,30], and no differences between sexes [31,32,33] have all been reported in the development of adverse cardiac effects following DOX chemotherapy. In the adult cancer patient population, with age range often spanning >50 years in studies reporting on sex as a predictor of cardiac events, age diversity complicates the interpretation based on increased risk of cardiac dysfunction with advancing age. Nevertheless, when sex differences are present, data suggests that adult males are generally at a greater risk for the development of cardiac events [34,35,36,37]. Finally, when evaluating sex differences in an exclusively elderly population (≥65 years old), no differences between sexes were revealed [38]. Although the precise factors that affect susceptibility between sexes over the lifespan remain unclear, it is hypothesized that menopausal state and concurrent changes in sex hormones may play a significant role [9].

#### 2.1.2. Sex-Related Differences in the Development of Cardiotoxicity: Preclinical Perspective

Preclinical evaluation of DOX cardiotoxicity allows for the removal of numerous confounding variables to aid in the understanding of risk factors associated with the development of adverse cardiac events. To date, few studies have directly compared sexual dimorphism in the development of cardiac dysfunction when DOX treatment is initiated in juvenile rodents [39,40]. Longo et al. delivered an 18 mg/kg cumulative dose of DOX at 14 days of age and reported similar reductions in cardiac mass between sexes upon euthanasia [39]. A separate study by Zeiss et al. utilized age-matched male and female mice that received a total of 25 mg/kg DOX [40]. In this study, ten distinct strains of mice were utilized, with each demonstrating some degree of cardiac damage. Six strains exhibited similar impairments between sexes, three strains showed greater cardiac injury in males, and one strain demonstrated greater compromise in females [40]. In adult rodent models, when compared independently, DOX-induced cardiac dysfunction has been demonstrated in both males and females [41,42,43]. However, similar to adult cancer patients, preclinical evidence in rodents suggests that males are more affected than females following DOX exposure [44,45]. Several features of DOX-induced cardiomyopathy that are more pronounced in male rats compared to females include cardiac atrophy, reduced left ventricle ejection fraction (LVEF), myocardial fibrosis, and myolysis [45]. Early studies also revealed that DOX treatment resulted in higher toxicity in 24-month-old animals when compared with six-month-old animals [46], representing an increase in DOX cardiotoxicity with age; however, sex differences in the development of cardiotoxicity in aged animals remain unexplored. Similar to patients, sex differences in the incidence and severity of DOX-induced cardiac dysfunction in rodents at any age could be an effect of reproductive hormone levels, as estrogen appears to affect sensitivity to DOX cardiac damage in female rats [47,48,49]. While additional work is needed to precisely establish sex-based differences, current evaluation reveals distinct similarities in the development of cardiac damage between clinical and basic science reports.

### 2.2. Clinical Manifestation of Doxorubicin-Induced Skeletal Muscle Weakness

Deficits in physical performance are a hallmark of skeletal muscle fatigue following DOX treatment, with limitations in many patients manifesting as reduced exercise capacity, muscle strength, and motor coordination [8,50]. Symptoms of fatigue are reported to worsen throughout the duration of the chemotherapy regimen and can persist for months or years following cessation of treatment [51,52,53,54]. Most commonly, fatigue is assessed via questionnaire, where patients report deviations in physical quality of life based on their perceived functional capacity [55]. Changes in VO_2_ peak and 6- or 12-min walk distance are also used to determine exercise intolerance and to diagnose exertional dyspnea in patients treated with DOX [56,57]. Furthermore, it is well-known that skeletal muscle exposure to DOX results in catabolism, where direct interaction with myofibers results in loss of muscle mass [58,59,60].

#### 2.2.1. Sex-Related Differences in the Development of Skeletal Muscle Weakness: Clinical Perspective

Fatigue that occurs during chemotherapy is described as a distressing and pervasive symptom with physical, mental, and emotional components characterized by a lack of energy [61]. Work focused on delineating disparities in symptoms of fatigue between pediatric and adult cancer patients led to the development of age-specific fatigue scales that are based on actual patient experiences [61,62]. Use of the Childhood Fatigue Scale and Adolescent Fatigue Scale in a group of pediatric cancer patients, led to the finding that self-reported fatigue in patients aged 7–18 was greater following DOX chemotherapy compared to cisplatin or ifosfamide [61]. Assessment of fatigue in two independent studies that included DOX as a component of their treatment protocol reported the manifestation of chemotherapy-related fatigue in patients 2–18 years of age, with no influence of sex [63,64].

Reports of sex differences for chemotherapy-related fatigue in adults revealed either no differences or higher fatigue scores for female cancer patients [65]. An important factor to consider when interpreting these results is that in general, women have been shown to report higher rates of symptoms than men [66]. In regard to functional outcomes, when compared to age and sex matched controls, male and female survivors of acute lymphoblastic leukemia are able to perform most basic motor functions. However, musculoskeletal morbidity and motor ineptness are present in survivors of both sexes, and females exhibit worse passive ankle dorsiflexion range of motion compared to males [67]. In addition, changes in musculoskeletal function can influence exercise capacity, and female patients exposed to DOX exhibited increased exercise intolerance and impaired aerobic capacity compared to males [51]. Finally, direct skeletal muscle damage has been demonstrated following hyperthermic isolated-limb perfusion, with this treatment generally revealing greater toxicity in females, which is attributed to a lower muscle to fat ratio [68]. Comparison of muscle biopsies from a cohort of five adult patients pre- and post-hyperthermic isolated limb perfusion using DOX specifically revealed muscle atrophy in both sexes, but patient sample size was insufficient to determine if precise differences exist between males and females [58]. Currently, there is a paucity of clinical reports evaluating DOX-related skeletal muscle weakness and fatigue. Extensive work is needed to expand the clinical evaluation of DOX-induced skeletal muscle toxicity, and sex differences need to be considered as an important variable in future studies in this field.

#### 2.2.2. Sex-Related Differences in the Development of Skeletal Muscle Weakness: Preclinical Perspective

The effect of DOX on skeletal muscle in a preclinical model was first published in 1985, when Doroshow et al. showed dramatic ultrastructural damage to the diaphragm following intraperitoneal injection in male mice [69]. Subsequent studies have corroborated this evidence and demonstrated atrophy and contractile dysfunction within both fast- and slow-twitch muscles [1]. While evidence in both male [43,70] and female [71,72] models of DOX myotoxicity demonstrates skeletal muscle atrophy and dysfunction, to date, no study has directly evaluated differences between the sexes when standardized for age, dose of DOX, and rodent strain. Meta-analysis of preclinical studies focused on the effects of DOX on skeletal muscle found male bias within the literature, as the predominance of studies have been conducted in male rodents [73]. Accordingly, preclinical comparison between males and females is still needed to establish if specific sex differences exist in the development and presentation of skeletal muscle weakness and fatigue.

## 3. Mechanisms for Sex Differences in Doxorubicin-Induced Myotoxicity

**Figure 2:**
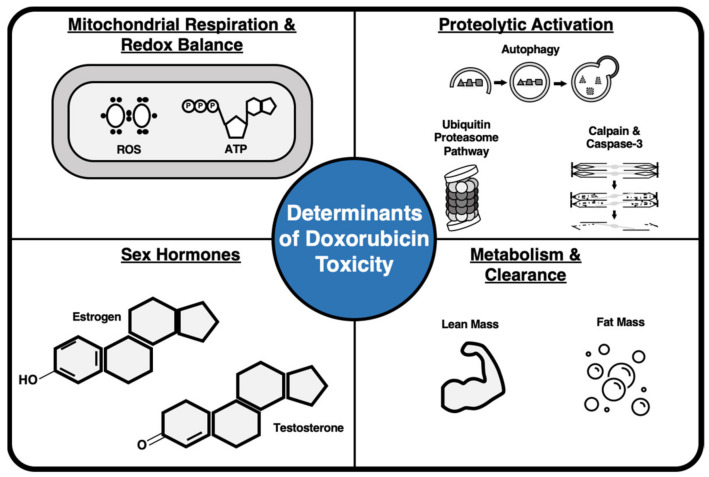
Factors associated with the development of doxorubicin myotoxicity in males and females include impaired mitochondrial respiration and redox imbalance, enhanced proteolytic breakdown of cardiac and skeletal muscle tissue, concentration of sex hormones, and compromised metabolism and clearance.

The physiological adaptations that mediate sexual dimorphism in the development of DOX-induced myotoxicity are not well understood. Mechanisms hypothesized to account for disparities between sexes in the presentation of cardiac and skeletal muscle dysfunction include differential regulation of redox balance, proteolytic activation, variation in sex hormones, and deviation in DOX clearance (Figure 2). The following sections will examine current literature in both clinical and preclinical populations to establish the relevance of each mechanism to the development of DOX-induced cardiac and skeletal muscle toxicity in either sex.

### 3.1. Doxorubicin-Induced Redox Imbalance

DOX-induced myotoxicity is characterized by redox imbalance and consequent impairment in cell signaling and function, which occurs due to supraphysiological reactive oxygen species (ROS) generation [74,75]. DOX increases cellular ROS production via (1) a one-electron reduction of DOX, forming a semiquinone that can reduce oxygen; (2) metabolic turnover of DOX, which forms highly reactive intermediates and products; and (3) direct interaction between DOX and iron or other metal ions [74,76]. Pharmacokinetics of DOX widely demonstrate a high affinity of DOX for cardiolipin, resulting in localization at mitochondrial complex I [77,78,79]. Ultimately, overproduction of ROS within mitochondria can induce mitochondrial DNA mutations, damage components of the mitochondrial respiratory chain, alter membrane permeability, influence mitochondrial calcium homeostasis, and reduce antioxidant defense systems [80]. It has been established that mitochondrial function plays a critical role in DOX-induced cardiac and skeletal muscle toxicity and, notably, targeting mitochondrial ROS pharmacologically prevents the DOX-induced cardiac and skeletal muscle abnormalities [6,41].

#### 3.1.1. Sex Differences in Redox Abnormalities and Mitochondrial Respiration with Doxorubicin Treatment

Fundamental differences exist in mitochondrial biology between males and females, independent of DOX [81]. Specifically, in the rodent heart, females demonstrate a lower mitochondrial content, but a more efficient phenotype that produces less ROS compared to males [81,82,83]. In addition, a review by Ventura-Clapier et al. concluded that skeletal muscle mitochondria in males versus females differ in oxidative capacity, resistance to oxidative stress, and calcium handling [81]. Indeed, mitochondrial DNA, mitochondrial protein content, and oxidative phosphorylation were shown to be higher in females and correlated with higher endurance exercise capacities [84,85,86].

Despite likely inherent sex differences in mitochondria, only two studies have directly investigated variations in DOX-induced myocardial mitochondria abnormalities between males and females [45,87]. The first, published in 2004 by Jang et al., revealed that males produce more mitochondrial hydrogen peroxide than females acutely following DOX administration, and that no differences exist in mitochondrial oxygen consumption between sexes [87]. In contrast, Moulin et al. showed that the mitochondrial respiration rate was significantly reduced following DOX administration, but only in male rats [45]. Thus, these preclinical observations suggest that altered mitochondrial function may partially account for the sexual dimorphism in DOX cardiotoxicity.

The paucity of preclinical studies directly comparing DOX effects on cardiac and skeletal muscle mitochondria in males and females necessitates additional comparison across studies for inference of potential sex differences. In this regard, data from both female and male rodent models demonstrate that DOX treatment results in increased lipid peroxidation, hydrogen peroxide emission, and mitochondrial dysfunction [6,42,88,89,90,91,92]. However, increased vulnerability to DOX-induced cardiomyopathy in male rodents is associated with mitochondrial lesions and aberrant changes in oxidative stress gene expression, suggesting that cardiac redox imbalance may be more severe in males compared to females [44,45,93,94,95]. In skeletal muscle, findings across studies suggest that both sexes demonstrate increased skeletal muscle oxidative stress and impaired mitochondrial respiration in response to DOX treatment [6,7,41,42,71,96,97]. Whether there are sex differences in the degree of this DOX-induced increase in oxidants and compromised respiration in skeletal muscle remains to be tested in studies designed specifically for that purpose.

#### 3.1.2. Sex-Specific Effects on Redox-Sensitive Proteolytic Systems in Cardiac and Skeletal Muscle

Cellular proteolytic systems are partially regulated by redox status [98,99,100]. Evidence suggests that DOX treatment results in increased activity of the major proteolytic pathways (i.e., ubiquitin proteasome pathway, autophagy, calpain, and caspase-3) [6,101,102,103,104,105]. Although it was previously highlighted that males are potentially more vulnerable to mitochondrial insult following DOX treatment, particularly in cardiac tissue, there do not appear to be sex differences in the extent of consequent proteolytic pathway activation. Both male and female animals treated with DOX showed a significant increase in key components of the ubiquitin proteasome pathway, such as MuRF-1 and atrogin-1/MAFbx, in cardiac and skeletal muscle [41,43,106,107,108]. Upregulated autophagy signaling has also been reported in both sexes, with cardiac and skeletal muscle tissue from DOX-treated animals showing increases in markers of autophagosome formation [41,109,110]. Similarly, caspase-3 and calpain are upregulated in cardiac and skeletal muscle isolated from both male and female rodents treated with DOX [6,87,97,107,111,112]. Thus, DOX appears to universally activate these degradative systems in males and females, but revealing sex differences in the degree of this upregulation will require additional studies.

### 3.2. Variation in Sex Hormones

Clinical evidence of sex differences in DOX myotoxicity may be a consequence of lifecycle variation in sex hormone levels and are unique to the age of the patient population studied. In women, cardiac muscle is largely protected from a variety of insults when estrogen levels are high, with the ratio of testosterone to estrogen predictive of cardiovascular disease (CVD) risk [113,114]. In contrast, low testosterone levels in men increases mortality from CVD and conversion of testosterone to estradiol may be essential for its protective effects [113]. In skeletal muscle, reduced sex hormone production is believed to contribute to age-induced decreases in strength and power [115,116]. The gradual decline in estradiol production in women and testosterone in men beginning near middle age is associated with the onset of muscle weakness. This relationship has been demonstrated clinically as hormone replacement therapy in both men and women is associated with improved muscle strength and function [117]. Overall, these findings support the hypothesis that sex-specific levels of sex hormones affect the incidence of CVD and muscle weakness throughout the lifespan.

#### Contribution of Sex Hormones to the Development of Doxorubicin Myotoxicity

Sexual dimorphism in the manifestation of DOX cardiomyopathy in male and female cancer patients follows age-related fluctuations in reproductive hormones. Relatively low levels of estrogen in pre-pubertal and post-menopausal women occur alongside these patients’ increased vulnerability to DOX cardiotoxicity compared to age-matched men [25,26,118]. Indeed, in pre-pubertal patients, the risk factors of female sex and cumulative DOX dose interact, meaning that the probability of cardiac events is greater in DOX-treated girls than boys and that this sex difference intensifies at higher DOX doses [25]. In older patients, clinical data showing the increased likelihood of adverse cardiac events in anthracycline-treated breast cancer patients who are post-menopausal versus pre-menopausal similarly highlight the potential protective role of estrogens in DOX-induced cardiotoxicity [118].

The ability of reproductive hormones to influence DOX myotoxicity may stem from their modulation of cardiac and skeletal muscle redox status [119,120]. Indeed, estrogen has been shown to enhance respiration, mitochondrial membrane fluidity, and antioxidant capacity [121,122]. These actions may explain why ovariectomized (OVX) rodents exhibit exacerbated DOX cardiotoxicity compared to DOX-treated cycling females [44]. Further supporting the protective role of estrogen in DOX cardiotoxicity are data showing the attenuation of DOX cardiac effects in OVX rodents following supplementation with estrogen [48,123]. The role of testosterone in DOX-induced cardiotoxicity, however, remains unclear, with studies suggesting exacerbated cardiotoxicity [94], no change in cardiac function [48], or attenuation of DOX-induced senescence [124] and oxidative stress [125]. For DOX-induced skeletal muscle toxicity, studies are needed to determine the effects of varying sex hormone levels.

### 3.3. Mechanisms of Doxorubicin Metabolism and Clearance

Dose calculation for DOX is based on patient body surface area and relies on the notion that clearance through the hepatic and renal system is related to body size [126,127,128]. However, differences in body composition between patients may play a large role in affecting DOX pharmacokinetic parameters, as the ratio of fat-free mass to fat-mass in patients with identical body surface area scores can vary drastically. In general, DOX is extracted from the circulation by the liver and then excreted into the bile, with a smaller portion of the drug undergoing renal clearance and exiting the body in urine [129,130,131]. Variability in DOX clearance may not only diminish its antineoplastic efficacy but also potentially exacerbate off-target toxic effects due to impaired drug metabolism and DOX accumulation in healthy tissues [2].

#### Sex Differences in Doxorubicin Metabolism and Clearance

Clinical investigation into the sex differences of DOX pharmacokinetics suggests that men have higher DOX clearance rates than women, and that individuals with breast cancer may have slower clearing of the drug [132]. Indeed, evidence suggests that excretion rate decreases with increased adiposity [133,134], which is significant because women tend to have greater percent fat mass than men [135,136,137]. These data support the notion that basing DOX dosing on patient body surface area may contribute to sex-specific differences in DOX toxicity and that body surface area is a poor marker of body composition in cancer patients, especially within comparable age groups [133,138]. Furthermore, DOX does not accumulate to a great extent in the fat tissue itself, and therefore, has the ability to travel to and influence other tissue types, such as the heart or skeletal muscle [25,26]. The sex differences in DOX distribution in pediatric patients may be explained due to the higher fat mass in girls than boys, thus contributing to the accumulation of DOX in off-target tissue types [25,26]. In regard to the role of obesity in DOX clearance, it is interesting to consider that excretion of DOX through the bile is the drug’s main route of clearance, and bile acid metabolism is significantly impaired in obesity [139,140]. Therefore, obesity-related abnormalities in bile acid synthesis or transport may play a role in impaired DOX clearance. Continued investigation into the mechanisms by which adipose tissue controls DOX clearance, potentially through secreted factors, may reveal sex-specific patterns in DOX action at cardiac and skeletal muscle.

## 4. Exercise Training as a Therapeutic Countermeasure for DOX-Induced Myotoxicity

Clinical evaluation of exercise as a novel therapy to improve cancer patient functional capacity was founded in the 1980s [141], with subsequent assessment of exercise training demonstrating clinical and preclinical success as a countermeasure to reduce cardiac pathology and muscular fatigue following DOX treatment specifically [142,143,144,145,146,147]. Clinical exercise interventions have confirmed the feasibility, safety, and efficacy of incorporating exercise training as standard treatment for cancer patients to ameliorate treatment-related adverse effects and to promote overall health benefits [148]. Additionally, rodent studies have provided insight into the molecular mechanisms for the beneficial effects of exercise to combat DOX myotoxicity [1]. These reports support the concept that exercise is capable of improving or preserving muscle function and quality of life through the upregulation of cellular pathways with the potential to limit mitochondrial dysfunction and ROS production in muscle tissue [149]. This section will summarize the current evidence regarding sex-specific exercise training adaptations to mitigate DOX-induced myotoxicity.

### 4.1. Sex-Related Differences in Muscular Adaptations to Doxorubicin Following Exercise Training: Clinical Perspective

**Figure 3:**
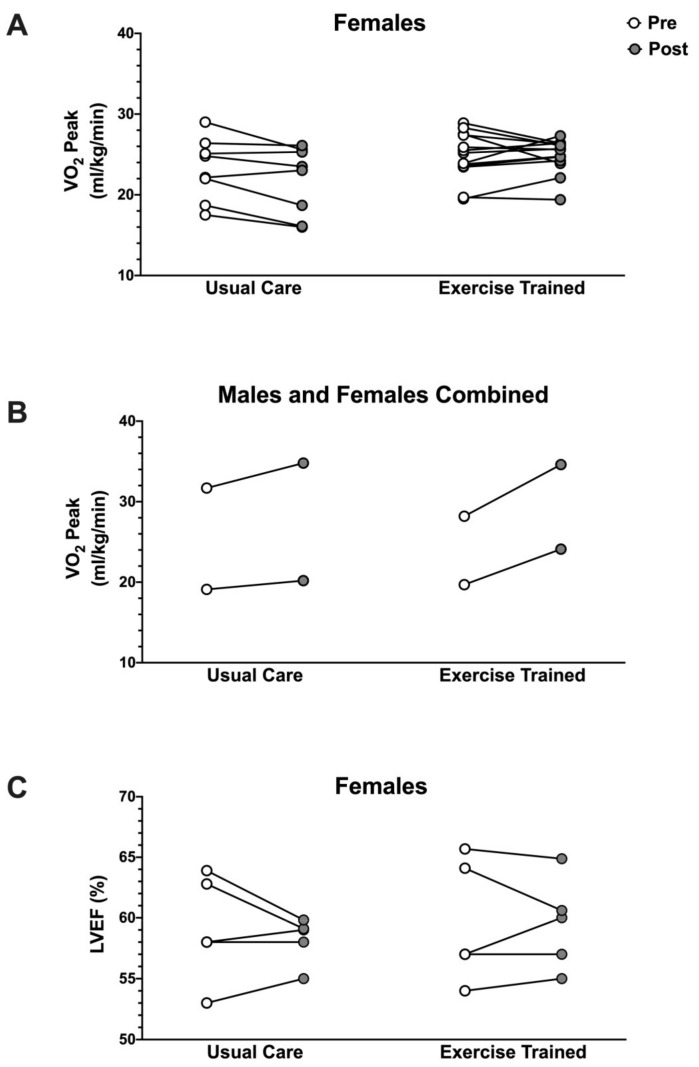
Exercise outcomes in cancer patients. (**A**) Change in VO_2_ peak in female breast patients undergoing either usual care or an exercise training intervention during chemotherapy treatment [57,143,152,153,155,156,157,158,159]. (**B**) Change in VO_2_ peak in mixed sex cancer patients undergoing either usual care or an exercise training intervention during chemotherapy treatment [160,161]. (**C**) Change in left ventricle ejection fraction (LVEF) in female breast patients undergoing either usual care or an exercise training intervention during chemotherapy treatment [57,143,153,166,167,168].

The beneficial effects of exercise against cardiotoxicity in male and female cancer patients are widely recognized [150]. Unfortunately, the majority of studies have not addressed sex as a variable in the study design. In addition, there is also a large disparity in the proportion of studies focused on males versus females, with the majority dedicated to female breast cancer patients [151]. Within studies where DOX is utilized as part of the chemotherapy regimen, there is a general consensus that exercise performed more frequently and at higher intensities results in greater benefits to breast cancer patients [143,152,153,154]. Indeed, the first study to assess cardiac function in female breast cancer patients receiving DOX concluded that aerobic training prescriptions that incorporate high-intensity interval training improve cardiac function during neoadjuvant chemotherapy [143]. Further, when VO_2_ peak is evaluated as a marker of cardiorespiratory fitness and exercise capacity in female patients, decrements are more frequently observed in the usual care groups, while exercise-trained groups maintain or have less severe reductions in VO_2_ peak [57,143,152,153,155,156,157,158,159]. Interestingly, although evidence is limited, reports that include both male and female participants show that VO_2_ peak is maintained in the usual care groups and is by comparison drastically increased in exercising groups [160,161] (Figure 3). Thus, despite no studies evaluating the male-specific response to DOX plus exercise training, these combined patient data may reveal a sex difference nonetheless. Indeed, the promising increases to cardiorespiratory fitness shown when the sexes are combined contrast the lesser improvements described for studies of exclusively female patients. It is interesting to consider that greater cardiac-related benefits for males undergoing exercise training in combination with DOX chemotherapy may be behind this discrepancy.

In regard to skeletal muscle function and quality of life, randomized clinical trials have tested the effects of exercise to reduce chemotherapy-related fatigue and improve patients’ ability to perform activities of daily living [151]. A recent meta-analysis comparing 113 of these studies found that exercise and the combination of exercise with psychological interventions reduce cancer-related fatigue during and after cancer treatment [151]. From these studies, 11,525 unique participants were compared, with the majority being female (78%) and the principal cancer type breast cancer (46.9%) [151]. Importantly, evaluation of sex differences in treatment effectiveness within this meta-analysis revealed no differences between sexes [151]. Consideration of age as an independent predictor of patient improvements also showed no influence on intervention efficacy (median age 54; range 35–72) [151]. Moreover, a recent study looking at exercise as a rehabilitative tool showed that cancer survivors that participated in a 12-week exercise-based oncology rehabilitation program had increased physical performance and reduced fatigue [162]. However, analysis of sex differences revealed that male participants had greater improvements in distance covered during a 6-min walk test and power production compared to females [162].

Although current research suggests that males may obtain a greater positive response to exercise training than females, several factors should be considered in future study design. Particularly, additional biomarkers and measurements need to be incorporated when assessing muscle functional outcomes. Typically, measurement of LVEF is performed as a primary marker of cardiac function and relative VO_2_ peak as a predictor of left ventricular function, CVD risk, quality of life, and fatigue [163]. However, the majority of reports concluding that exercise improves physical functioning in cancer patients receiving DOX chemotherapy tend to show no statistical difference in these factors (Figure 3). This could potentially occur for several reasons, such as study inclusion criteria, which often select cancer patients with no known pre-existing conditions and eliminate patients most at risk for DOX toxicity, lack of individualized exercise therapy to optimize patient benefits, and/or study timelines where patients are only monitored through treatment and long-term results remain unknown [164]. In addition, Kirkham et al. suggest monitoring hemodynamic response to exercise and DOX rather than changes in LVEF and VO_2_ peak [153]. This conclusion is logical, as impaired endothelium-dependent dilation is a key initial step in the pathogenesis of CVD [165]. Furthermore, patient awareness of their group assignment may itself create unintended bias, as patient perceived benefits of exercise may have significant placebo effects. For example, a randomized clinical trial comparing aerobic exercise training versus usual care showed positive changes in hemodynamics, musculoskeletal symptoms, mood, and body weight only in the exercise group, although there was no significant difference in the duration of moderate-to-vigorous physical activity performed per week between groups [166]. Thus, additional work is needed to fully elucidate the degree of sexual dimorphism between cancer patients and survivors undergoing exercise training.

### 4.2. Sex-Related Differences in Muscular Adaptations to Doxorubicin Following Exercise Training: Preclinical Perspective

The protective effects of exercise against DOX cardiac and skeletal muscle toxicity are established from independent observations in both male and female rodents [1]. While no exercise study has directly determined if sex differences exist in the development of cardiac dysfunction following DOX exposure, separate publications by the same research group reported a similar reduction in fractional shortening in male (−22%) and female (−24%) rats that received a 10mg/kg bolus intraperitoneal dose of DOX [169,170]. Additionally, this group reported that 10 weeks of treadmill or voluntary wheel running prior to administration of DOX significantly increased fractional shortening in both male and female rats compared to their sedentary counterparts [170,171]. Investigation into the effects of exercise training on DOX cardiotoxicity in tumor-bearing rodents also highlights the protective effects of exercise [172,173,174]. Although the individual study designs vary greatly, their results emphasize the efficacy of exercise as a therapy to combat DOX cardiotoxicity, with no apparent detrimental effects on DOX antineoplastic activity in male or female rodents [172,173,174].

Fewer studies exist evaluating the effects of exercise on DOX-induced skeletal muscle pathology, with greater variation between studies due to the specific muscle evaluated. Preclinical studies of DOX skeletal muscle myopathy typically focus on the soleus and extensor digitorum longus muscle, with reports also implicating defects to diaphragm muscle function [42,175]. To date, no rodent studies investigating exercise and DOX myotoxicity have directly evaluated sex differences; however, current evidence suggests that skeletal muscle morphology and function are improved in both males and females exposed to exercise training [42,72,175,176,177].

Mechanisms associated with the protective effects of exercise against DOX cardiac and skeletal muscle toxicity in both male and female rodents appear related to preclusion of several determinants of DOX myotoxicity. Specifically, independent evaluation of mitochondrial function in male and female rats exposed to 1–2 weeks of exercise conditioning prior to DOX treatment revealed a cardiac mitochondrial phenotype that was protected from DOX-induced impairments in mitochondrial oxygen consumption and supraphysiological mitochondrial ROS production [42,178]. Indeed, even a singular bout of exercise is protective against DOX-induced myocardial lipid peroxidation [179] and regulates superoxide dismutase activity in male [180] and female [181] rats. Preservation of mitochondrial oxidative respiration and redox balance in the heart and soleus of DOX treated rats with exercise training mitigated MuRF1 signaling in male rats but not Atrogin-1/MAFbx [43]. In contrast, exercise training in female OVX rats did not protect against aberrant MuRF1 activation and showed no change in atrogin-1/MAFbx signaling [177]. However, DOX-induced autophagy signaling and activation of calpain and caspase-3 is attenuated in both male and female rats with exercise [97,109,110,178,182,183]. Interestingly, when exercise is integrated into the OVX model, the cardioprotective effects may be equal to or surpass that of estrogen supplementation alone [184]. A separate study in male rats purports that endurance exercise training attenuates cardiac dysfunction caused by androgen deprivation therapy during DOX treatment [185]. Finally, cardiac and skeletal muscle accumulation of DOX is also decreased in the heart and skeletal muscles of DOX treated male and female rodents following exercise training [42,170,174,186]. Therefore, positive modifications to mitochondrial metabolism and DOX clearance from muscle tissue may play an important role in modulating exercise-induced myoprotection following DOX exposure. Future studies are needed to control for experimental variables (i.e., DOX dosing strategy, exercise prescription, muscles evaluated, etc.) to precisely determine if sexual dimorphism exists in the therapeutic potential for exercise training to reduce DOX myotoxicity.

## 5. Conclusions

Given the clinical utility of DOX as an antineoplastic agent, it is important to determine the factors responsible for its off-target toxicity. Enhanced understanding of the factors that contribute to DOX myotoxicity could increase its safety profile by limiting patient dose for those with greater established risk for adverse side effects. In this regard, evidence currently exists for sex differences in the development of DOX myotoxicity in the patient population based on differences in age, sex hormones, and body composition. Additionally, participation in regular bouts of exercise provides beneficial adaptations to both males and females, which help to maintain cardiovascular health and reduce fatigue by improving mitochondrial function and reducing the accumulation of DOX within the heart and skeletal muscle. However, continuing work is needed to improve study design and outcome measures to fully elucidate the factors affecting sexual dimorphism in DOX-induced myotoxicity.
